# Delineating the intra-patient heterogeneity of molecular alterations in treatment-naïve colorectal cancer with peritoneal carcinomatosis

**DOI:** 10.1038/s41379-022-01012-y

**Published:** 2022-02-15

**Authors:** Christina Siesing, Alexandra Petersson, Thora Ulfarsdottir, Subhayan Chattopadhyay, Björn Nodin, Jakob Eberhard, Jenny Brändstedt, Ingvar Syk, David Gisselsson, Karin Jirström

**Affiliations:** 1 Division of Oncology and Therapeutic Pathology, Department of Clinical Sciences Lund, Lund University, Lund, Sweden; 2 Department of Oncology, Skåne University Hospital, Lund, Sweden; 3 Division of Clinical Genetics, Department of Laboratory Medicine, Lund University, Lund, Sweden; 4 Division of Surgery, Department of Clinical Sciences Malmö, Lund University, Malmö, Sweden

**Keywords:** Colorectal cancer, Tumour heterogeneity

## Abstract

In a non-negligible number of patients with metastatic colorectal cancer (mCRC), the peritoneum is the predominant site of dissemination. Cure can be achieved by cytoreductive surgery (CRS) and hyperthermic intraperitoneal chemotherapy (HIPEC), but this procedure is associated with long-term morbidity and high relapse rates. Thus, there is a pressing need for improved therapeutic strategies and complementary biomarkers. The present study explored the molecular heterogeneity in mCRC with peritoneal carcinomatosis (PC), and the potential clinical implications thereof. Multi-region immunohistochemical profiling and deep targeted DNA-sequencing was performed on chemotherapy-naïve tumours from seven patients with synchronous colorectal PC who underwent CRS and HIPEC. In total, 88 samples (5-19 per patient) were analysed, representing primary tumour, lymph node metastases, tumour deposits, PC and liver metastases. Expression of special AT-rich sequence-binding protein 2 (SATB2), a marker of colorectal lineage, was lacking in the majority of cases, and a conspicuous intra-patient heterogeneity was denoted for expression of the proposed prognostic and predictive biomarker RNA-binding motif protein 3 (RBM3). Loss of mismatch repair proteins MLH1 and PSM2, observed in one case, was concordant with microsatellite instability and the highest tumour mutational burden. When present in a patient, mutations in key CRC driver genes, i.e., *KRAS, APC* and *TP53*, were homogenously distributed across all samples, while less common mutations were more heterogenous. On the same note, copy number variations showed intra-patient as well inter-patient heterogeneity. In two out of seven cases, hierarchical clustering revealed that samples from the PC and lymph node metastases were more similar to each other than to the primary tumour. In summary, these findings should encourage additional studies addressing the potential distinctiveness of mCRC with PC, which might pave the way for improved personalized treatment of these patients.

## Introduction

Although lymphogenic and hematogenic dissemination are the dominant pathways of distant metastasis in colorectal cancer (CRC), a non-negligible number of patients are affected by cancer cell seeding into the peritoneal cavity, causing peritoneal carcinomatosis (PC). Studies have reported a prevalence of 4–13% of synchronous PC^1–3^ in CRC. Right-sided tumours, advanced T stage and N stage and non-radical resection of the primary tumour are, among others, reported risk factors for PC. Both Segelman et al. and Jayne et al. have reported that the peritoneum is the sole site of metastasis in approximately 60% of patients with colorectal PC^1,3^. PC often has a considerably negative impact on the quality of life of these patients, causing problems with for example ascites and malnutrition.

For patients with localized PC, cure is possible through cytoreductive surgery (CRS), often combined with intraperitoneal chemotherapy. CRS in combination with intraperitoneal chemotherapy has been performed on patients with colorectal PC since the 1980s^4^. From start, the chemotherapy was given intraperitoneally for up to six days post-surgery, so called early postoperative intraperitoneal treatment (EPIC). Today, the patients most often receive hyperthermic intraperitoneal chemotherapy (HIPEC) during the surgical procedure. However, the procedure is extensive and causes high morbidity, with a 30-day mortality rate of 1–8%^5–7^. Although combined CRS and HIPEC have been proved to be associated with significantly improved survival to systemic chemotherapy only^8,9^ the additional gain of HIPEC to CRS is now under debate. The PRODIGE 7 trial reported no survival gain in a group treated with HIPEC compared to a group that underwent only CRS with a median overall survival (OS) of 41.2 months and 41.7 months, respectively^10^. Hence, colorectal PC is a significant clinical concern in that substantial problems can arise both if the patients are left untreated and by the treatment itself. Therefore, it is important to cautiously select the patients who will actually benefit from CRS and HIPEC. Today, the two most important factors for a successful CRS procedure is a low peritoneal carcinoma index (PCI) score and completeness of cytoreduction (CC)^11^, but there is a pressing need to identify better prognostic and predictive tools to achieve a more adequate patient selection. While several studies have characterised the molecular landscape in liver and lung metastases originating from CRC^12–14^, PC has been much less explored in this regard^15^. Further insight could therefore lead to improvements in the clinical management of these patients.

*RAS* and *BRAF* mutation status are the only molecular biomarkers included in current treatment algorithms for patients with metastatic CRC (mCRC), to determine their eligibility for cytoreductive or maintenance therapy with EGFR inhibitors^16^. Immune checkpoint blockade may also be an option in patients with non-resectable mCRC with deficient mismatch-repair (dMMR)/microsatellite instability (MSI)^17^, but biomarkers for prediction of response to standard chemotherapies are still lacking. Among many proposed biomarkers of chemosensitivity, high expression of the RNA and DNA-binding protein RNA-binding motif protein 3 (RBM3) has been shown to be associated with a prolonged survival and response to oxaliplatin in mCRC^18^, which is also in line with studies on ovarian^19^ and pancreatic^20^ cancer. Moreover, the special AT-rich sequence-binding protein 2 (SATB2), a nuclear matrix-associated protein with tissue-specific expression in the lower gastrointestinal tract, has been shown to correlate with a prolonged survival and improved response to irinotecan in mCRC^21^.

The clinical management of patients with mCRC is further challenged by tumour heterogeneity, not least with regard to the accuracy of biomarker analyses, which are generally performed on a single tumour sample, most often from the primary tumour^16^. Heterogeneity, occurring within the primary tumour (intratumour heterogeneity), within metastases (intrametastatic heterogeneity), or between metastases (intermetastatic heterogeneity), provides a hotbed of clonal diversity from which resistant clones can be enriched under the selective pressure from systemic therapies^22^. Several studies have described the occurrence of tumour heterogeneity and clonal evolution in mCRC, mainly in the context of liver metastasis, where seeding appears to occur mainly from the primary tumours, but in some cases also from lymph nodes or other distant metastases^23–25^. Meanwhile, the molecular heterogeneity in colorectal PC remains less explored. The process of metastasis in PC, titled *the peritoneal metastatic cascade*, is thought to differ from other types of distant dissemination and includes the four steps detachment of tumour cells, peritoneal transport of tumour cells, attachment to distant peritoneum and invasion into the subperitoneal space^26,27^.

The aim of this study was to gain a deeper understanding of the molecular heterogeneity and dissemination patterns of colorectal PC. To this end, multi-region profiling with targeted deep sequencing (TDS) was performed on chemo-naïve primary tumours and metastatic lesions in seven patients with colorectal adenocarcinoma and PC who underwent CRS and HIPEC.

## Material and methods

### Study cohort

**Fig. 1:**
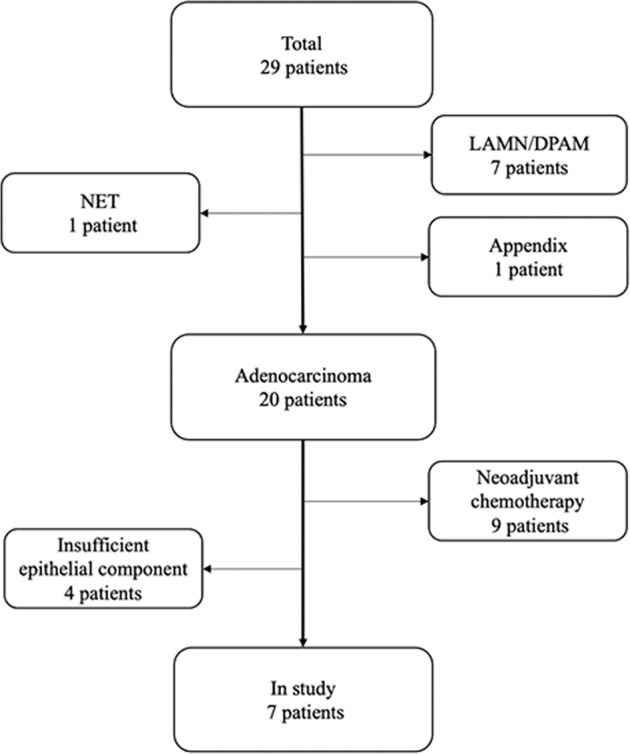
Flowchart of the patient selection. Flowchart showing the selection of patients from a local registry including all patients who underwent CRS in combination with HIPEC at Skane University Hospital, Malmö between January 1st 2013 and December 31st 2014. NET Neuroendocrine tumour, LAMN Low grade appendiceal mucinous neoplasm, DPAM Disseminated peritoneal adenomucinosis.

Cases were selected from a local registry of all patients who underwent CRS in combination with HIPEC at Skåne University Hospital in Malmö between January 1^st^ 2013 and Dec 31^st^ 2014. A total of 29 patients with primary tumours of colorectal origin with synchronous PC were identified and histopathologically re-evaluated, of which 20 cases were classified as primary colorectal adenocarcinoma. Nine cases who had received neoadjuvant chemotherapy were excluded, and in four of the remaining 11 cases, all with mucinous histology, the epithelial tumour component was deemed as being too sparse, leaving 7 cases eligible for the study (Fig. 1). Clinical data were retrieved from hospital records and information on distant metastasis, preoperative CEA level, date of surgery, Karnofsky performance scale index at time of surgery, cytoreduction score (CC), intraoperative chemotherapy agent, adjuvant chemotherapy treatment and recurrence were denoted. Follow up began at the time of surgery and ended at death or at 30^th^ of March 2021, whichever came first. Patient and tumour characteristics are shown in Table 1.

**Table 1:** Patient and tumour characteristics.

	Pat 1	Pat 2	Pat 3	Pat 4	Pat 5	Pat 6	Pat 7
Sex	F	M	F	F	M	M	F
Location of primary tumour	Right	Right	Left	Right	Rectum	Rectum	Right
T stage	3c	4b	4b	4a	4a	4a	4b
N stage	2b	2a	2b	2b	2b	2b	2b
Synchronous carcinosis	Y	Y	Y	Y	Y	Y	Y
Other distant metastases at diagnosis							Liver
PCI Score	8	13	8	24	13	4	9
CEA before surgery	231	10	2	-	3	3	73
Completeness of cytoreduction	0	0	0	0	0	0	0
IP treatment	EPIC	HIPEC	HIPEC	HIPEC	HIPEC	HIPEC	HIPEC
Histology	Signet-ring cell carcinoma	Adenocarcinoma	Mucinous adenocarcinoma	Adenocarcinoma	Adenocarcinoma*	Signet-ring cell carcinoma	Adenocarcinoma
Overall survival (months)	98	28	94	14	32	80	78
Vital status	1	0	1	0	0	1	1
Adjuvant chemotherapy	Y	Y	Y	N	Y	Y	Y
Number of samples IHC	53	52	41	69	70	50	68
Number of samples TDS	9	19	5	16	6	14	16

*Nearly equal distribution of adenocarcinoma and signet-ring cell carcinoma.

### Single patient tissue chip construction and immunohistochemistry

Histopathological re-evaluation of freshly sectioned haematoxylin and eosin-stained tissue slides from all cases was carried out by a senior pathologist (KJ). An individualised tissue microarray, a so-called single patient tissue chip (SPTC), was created for each patient as earlier described^28^. In brief, tissue cores of 1 mm in diameter were taken from multiple spatially divergent areas in each paraffin block containing tissue from the primary tumour. In addition, cores were taken from lymph node metastases, tumour deposits, defined according to the 7^th^ edition of the AJCC/TNM system as nodules >3 mm without any evidence of residual lymph node architecture, and peritoneal metastases of sufficient size, as well as from other metastatic sites if available^29^.

Normal mucosa from the colon, rectum or ileum was also sampled from each case. The cores were positioned in a recipient paraffin block using a semi-automated TMArrayer (Pathology Devices, Westminister, MD).

### Immunohistochemical staining and evaluation

For immunohistochemical (IHC) analysis of RBM3 and SATB2, 4 *μm* thick SPTC sections were automatically pre-treated with the PT -link system (Agilent Technologies, Glostrup, Denmark) and stained in an Autostainer Plus (Agilent Technologies) with monoclonal antibodies against RBM3 (clone AMAb90655, Atlas Antibodies, Bromma, Sweden, diluted 1:750), and SATB2 (clone AMAb90679, Atlas Antibodies, diluted 1:100). For IHC analysis of MMR proteins, 4 *μm* thick SPTC sections were automatically pretreated and stained using the Benchmark Ultra Ventana platform, with “ready to use” monoclonal antibodies against MLH1 (clone M1, Ventana/Roche, Basel, Schweiz), PMS2 (clone EPR3947, Ventana/Roche), and MSH2 (clone G219-1129, Ventana/Roche), and a monoclonal antibody against MSH6 (EPR3945, Nordic BioSite, Täby, Sweden, diluted 1:50).

All IHC evaluations were conducted independently by CS, TU, and KJ. The evaluators were blinded to clinical and outcome data. Differences in evaluation were then discussed to reach consensus. For RBM3 and SATB2, the estimated fraction (0%–100%) as well as the intensity (0 = negative,1 = weak, 2 = moderate, 3 = strong) of the nuclear staining was denoted for each individual tissue microarray (TMA) core. Samples from seminoma and normal colorectal mucosa, the latter included in the SPTC, were used as positive controls for RBM3 and SATB2, respectively. A histoscore was calculated by multiplying the fraction with the intensity, resulting in a score from 0 to 3. For MLH1, PMS2, MSH2, and MSH6, the fraction of positive nuclear staining was estimated for each individual TMA core (0%–100%). Stromal cells and lymphocytes in the background served as positive internal controls.

### DNA extraction

Along with the construction of the SPTC, an additional tissue core (1 mm diameter) was taken from areas immediately adjacent to selected SPTC-cores with a sufficient amount of tumour cells and from normal tissue, i.e., benign-appearing mucosa from the distal or proximal resection margins of the primary tumour specimen. DNA extraction was performed using the Qiagen Allprep FFPE DNA/RNA kit (Qiagen, Hilden, Germany) according to the manufacturer’s instructions.

### Targeted deep sequencing

A total number of 88 samples were selected for TDS, along with paired normal samples from each case (*n* = 7), using the INVIEW Oncopanel All-in-one (version 2.8) (Eurofins Genomics, Konstanz, Germany), which covers the entire exons of 591 cancer associated genes^30^. Hybridization-based target capture and library preparation was carried out using Agilent SureSelect technology (Agilent Technologies Inc, Santa Clara, CA, US) and sequencing was conducted with the NovaSeq 6000 S2 PE150 XP (Illumina, San Diego, CA, US). The bioinformatics analysis was performed by Eurofins Genomics using the Burrows-Wheeler Aligner (BWA, version 0.7.15^31^) with genome build hg38 (chronly, UCSC) as reference genome. PCR duplicates were excluded using sambamba (version .6.6^32^) and base quality recalibration was performed by GATK (version 3.7^33,34^) for all uniquely mapped on-target reads.

### Filtering of variants

Single nucleotide variants (SNVs), insertions and deletions (InDels) were detected in each sample using the variant caller LoFreq^35^. All variants were further filtered against the paired normal sample of each patient and were kept in downstream analyses based on read depth (≥100 x, following duplicate exclusion) and variant allelic frequency (VAF > 1%). Detected variants were also excluded if not present with a VAF ≥ 10% in one of the samples for that patient. To screen for variants with known clinical significance, the Catalogue of Somatic Mutations in Cancer (COSMIC, version 92^36^) database was utilized with selection for mutations with a primary histology annotated as “Large intestine” or “nonspecific” (NS) and classified as “Pathogenic”. An overview of the mutations and variant allele frequencies found in each case is shown in Supplementary Table 1.

### Copy number analysis

Copy number variations (CNVs) were detected using the caller CNVkit^37^. In brief, both on-target and off-target reads were used to designate the log2 copy ratios for each tumour sample. The combined log2 ratios were then normalized to the paired normal sample for each patient and further used to calculate the discrete copy number events. Only copy number events covered to a minimum of 100 reads were used for subsequent analyses.

### Tumour mutational burden

Tumour mutational burden (TMB) was calculated as a division of the total number of detected somatic coding variants by the targeted coding region size (Mb). Initially, all SNVs with a coverage depth above or equal to 50x and a VAF value above 5% were included in the calculation of the TMB. However, the TDS approach used in this study is directed towards genomic areas of interest in cancer development and in order to avoid selection bias, filtering was performed in concordance with previously published works^38,39^, with some exceptions; i.e., non-coding mutations, variants annotated as known somatic variants in the COSMIC (version 71^40^) and ClinVar^41^ databases, known constitutional variants according to dbSNP^42^, constitutional variants occurring at least twice in the ExAC (gnomAD) database^43^, predicted germline variants according to the somatic-germline-zygosity algorithm^44^ and mutations in tumour suppressor genes (Supplementary Table 2) were excluded.

### Microsatellite instability

MSI status was determined using the mSIGNS algorithm^45^. The number of diversely sized loci was quantified for each microsatellite locus and compared with a panel of normal controls. A locus was considered unstable if the number of repeats in the tumour sample was statistically significantly greater than in the controls. MSI status was determined by the ratio of unstable to stable loci. A ratio greater than 0.2 was considered MSI.

### Statistics

Spearman´s rank-order correlation analysis was used to investigate the relationship between MSI status and TMB. A *p*-value < 0.05 was considered statistically significant. Hierarchical clustering was performed using Euclidean distance measure to compute the distance matrix and Ward agglomeration method to obtain the clusters. The clustering is based on tumour specific mutations. The cophenetic correlation coefficient was used to evaluate the accuracy of clustering. The maximum value of the coefficient is 1 and a value > 0.75 was considered a good fit. Dendrograms were produced to visualize the clustering. All statistical analyses were performed using R (version 3.6.3) and RStudio (version 1.4.1103).

## Results

### Multiregional immunohistochemical profiling

**Fig. 2:**
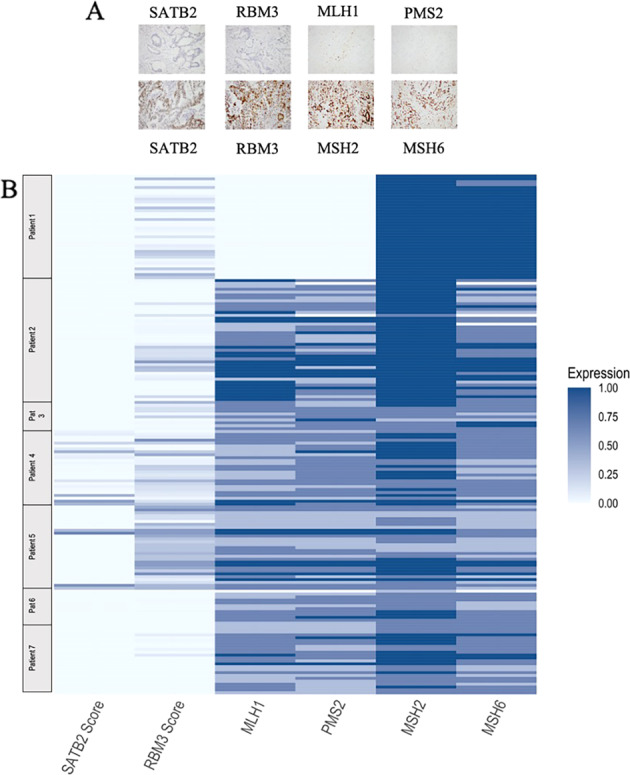
Sample immunohistochemical images and protein expression overview. **A** Sample immunohistochemical images, 20X magnification. From left to right: Negative and heterogenous SATB2 expression, Negative and strong RBM3 expression, Negative MLH1 expression, strong MSH2 expression, negative PMS2 expression, and strong MSH6 expression. **B** Heatmap illustrating IHC evaluation of protein expression for SATB2, RBM3, and MMR-proteins MLH1, PSM2, MSH2, and MSH6. Only samples where all proteins could be evaluated are shown.

Representative IHC images are shown in Fig. 2, together with a heatmap of the IHC expression of RBM3, SATB2, MLH1, PSM2, MSH2, and MSH6 (detailed view showed in Supplementary Table 3). Notably, SATB2 expression was all over low. The highest expression was seen in patient 4, mainly in lymph node metastases and peritoneal carcinomatosis. Patient 5 had heterogenous SATB2 expression with the highest expression seen in the primary tumour. SATB2 was denoted as completely negative in patients 1, 2, 3, 6, and 7. The overall strongest RBM3 expression was seen in the tumour tissue of patient 5. Patient 3 had a heterogenous expression of RBM3, with low expression in the lymph node metastases and a higher expression in the primary tumour and peritoneal metastases. A heterogenous, but allover lower, expression of RBM3 was also seen in patients 1 and 2, with several samples being RBM3 negative.

Patient 1 lacked expression of MMR-proteins MLH1 and PSM2 but displayed a consistently positive expression of MSH2 and MSH6. In all other cases, all four MMR-proteins were expressed with different degrees of heterogeneity. A more heterogenous expression of MLH1 and PSM2 was denoted in patients 3, 6, and 7 compared with the other cases. MSH2 was the most stably expressed protein in all cases.

### Spatial genomic profiling

**Fig. 3:**
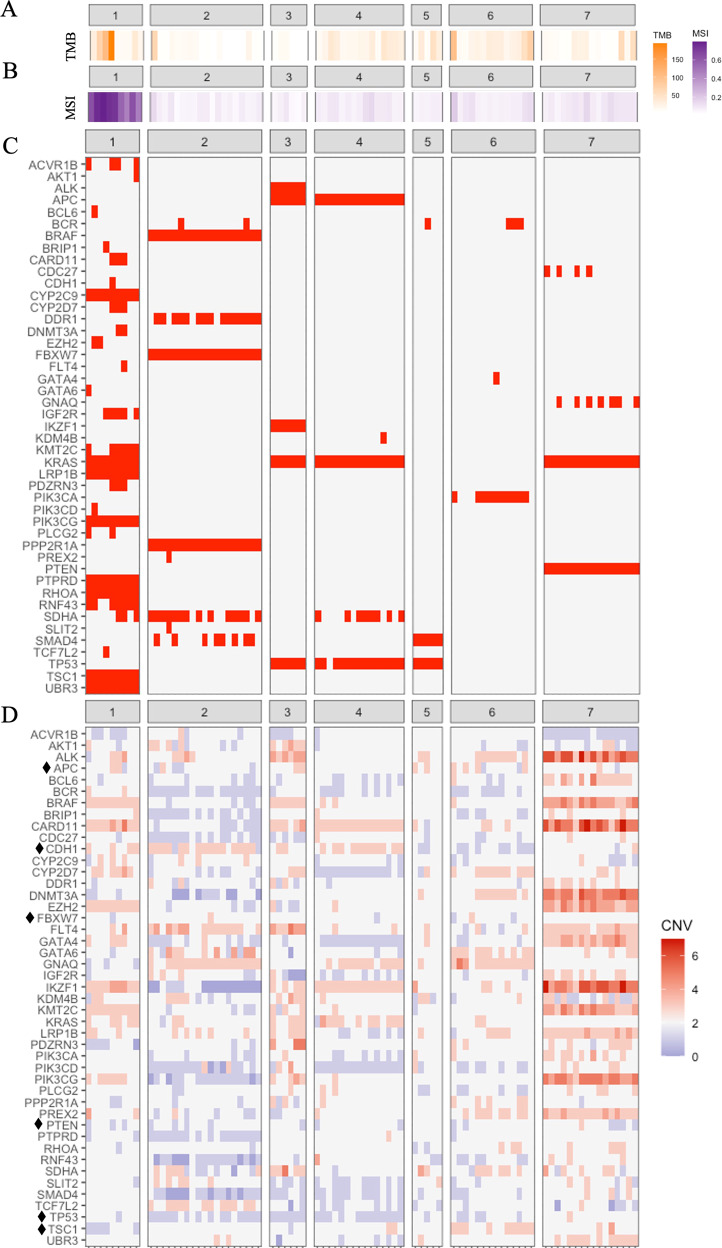
Genetic profiles. **A** Heatmap visualizing the TMB value as mutations per megabase. **B** Heatmap visualizing the MSI score as the ratio between unstable and stable loci. **C** Summary of genetic alterations (SNVs and InDels) of known clinical relevance detected in each tumour. Red = alteration. **D** Heatmap visualizing CNVs in the genes with genetic alterations showed in **C**, Grey = normal copy number, red = gains, blue = loss. Black diamond = tumour suppressor gene.

Out of the 88 tumour samples selected for genomic profiling, 4 were excluded from further analysis due to low DNA quality. The nature and anatomical location of all samples subjected to TDS analysis are shown in Supplementary Figure 1. In Patient 1, carcinomatosis was diagnosed on a biopsy taken during an initially planned laparoscopic surgery of the primary tumour, and this unexpected finding led to postponed surgery including CRS and EPIC. However, no further PC was found, and, hence, no PC sample was available for this study. The median number of samples from each patient was 14 (5–19 per patient). TMB values, MSI scores and altered genes with known clinical importance, together with CNVs detected covering any part of those genes, for each sample, are shown in Fig. 3. The highest TMB value was denoted in patient 1 (Fig. 3A), with a median value of 30.9 (range 10.5–193.4). In patients 4, 5 and 6, the median TMB values were 21.7, 28.5, and 26.5 mut/Mb, respectively (range 10.2–42.1; 8.8–50.1; 9.2–98.4) and in patients 2, 3, and 7, the TMB was low, with median values of 5.1, 4.1 and 9.0 mut/Mb (range 1.7–21.0; 1.02–1.2; 4.41–60.1), respectively.

All samples from patient 1 were denoted as MSI (Fig. 3B), with a median MSI score of 0.64 (range 0.46–0.80). Samples from all other patients were denoted as microsatellite stable (MSS), with a median MSI score between 0.06 and 0.09 (range 0.03–0.19). The highest variability was seen in patient 1, with an MSI score range of 0.33, and the lowest variability was seen in patient 5, with a range of 0.03. A moderate positive correlation was found between MSI-score and TMB (*r* = 0.44, *p*-value < 0.05).

Across all patients, *KRAS* was detected to be the most commonly altered gene (4/7), followed by *TP53* (3/7) and *APC* (2/7), as shown in Fig. 3C. Variants detected in all samples from a particular patient (shared) was found for all cases except one, patient 6. Shared mutations were noted in *CYP2C9, KRAS, LRP1B, PIK3CG, PTPRD, RHOA, TSC1,* and *UBR* in patient 1, *BRAF, FBXW7* and *PPP2R1A* in patient 2, *ALK, APC, IKZF1, KRAS* and *TP53* in patient 3, *APC* and *KRAS* in patient 4, *SMAD4* and *TP53* in patient 5 and *KRAS* and *PTEN* in patient 7. A ratio describing the proportion of shared versus the total number of detected clinically important alterations was calculated for each patient. The lowest ratio was seen in patient 6 (0) and the highest in patient 3 (1). The calculated ratio did not correlate to the number of samples included for each patient (*r* = 0.23, *p* = 0.61).

**Fig. 4:**
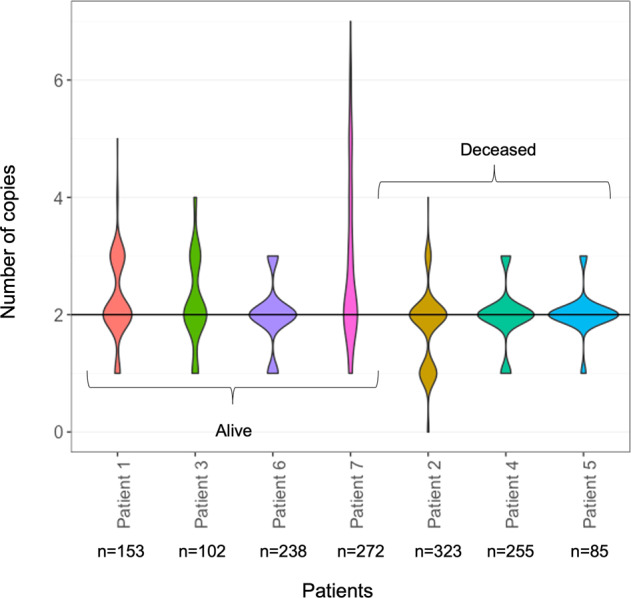
Visualization of copy numbers. Violin plot visualizing number of copies detected per patient. n number of observations included.

For all genes detected to be altered by a SNV or InDel (Fig. 3C), CNVs found to cover whole, or parts, of the gene sequence are shown in Fig. 3D. Across all samples, copy number gains were most commonly seen to involve regions including the genes *CARD11* (49/84), *IKZF1* (48/84), and *FLT4* (42/84). Heterozygous copy number losses were most commonly seen to affect *TP53* (39/84) and *PIK3CD* (37/84). The highest quantity of copy number losses was seen in patient 2, e.g., with homozygous loss of *IKZF1* and heterozygous loss of *BCR, SMAD4* and *TP53*. Of note, patients 3 and 4 had both an alteration (SNV or InDel) in and a heterozygous copy number loss of *TP53*. Furthermore, in addition to the noted inter-patient copy number heterogeneity, varying degrees of intra-patient heterogeneity were also observed within the patient cohort. This is for instance shown in samples originating from patient 7, where a duplication event was detected in some samples (3/16) within the *SMAD4* gene region, whereas an allelic deletion was found in one sample and where no copy number alteration was detected in the rest of the samples (12/16). As seen in Fig. 4, the highest number of copy number gains, both in terms of quantity and in allelic copy number, was denoted in patient 7, e.g., for *ALK* (median copy number 6, range 3–7), and *CARD11* (median copy number 5, range 3–7). Copy number gains were also frequently detected in genes other than in those found to contain SNVs or InDels, for example *GATA3* and *EGFR* (Table 2).

**Table 2:** Summary of genes with > 5 copy number gains but no mutations.

### Hierarchical clustering analyses

**Fig. 5:**
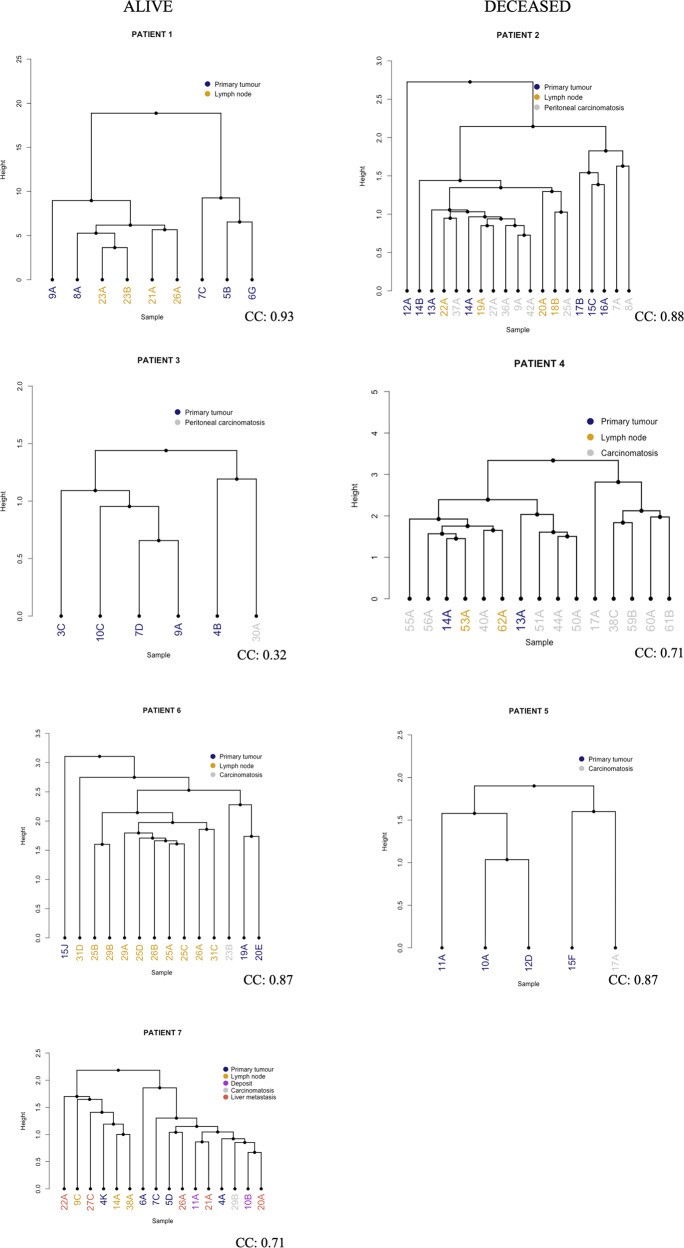
Hierarchical clustering. Dendrograms visualizing the hierarchical clustering for each of the seven patients included in the study. The dendrograms are sorted according to survival, with the dendrograms on the left including samples from patients who were alive at last follow-up and the dendrograms to the right including samples from patients who were deceased at last follow-up The leafs are colored after the nature of the samples. Blue circle indicates primary tumour, yellow circle indicates lymph node metastases, grey circle indicates peritoneal carcinomatosis, red circle indicates liver metastasis, purple circle indicates tumour deposit (possibly lymph node metastasis). CC Cophenetic coefficient.

The produced dendrograms for each patient are visualized in Fig. 5. The clustering for patient 1 revealed genetic similarities between the lymph node samples and primary tumour sample 8 A and 9 A, in contrast to the other primary tumour samples which formed a separate cluster. For patient 2, several PC samples (27 A, 36 A, 9 A, 42 A), clustered together with lymph node sample 19 A before they clustered with the primary tumour sample 14 A. PC sample 37 A and lymph node sample 22 A also clustered together before clustering with primary tumour sample 14 A. In patient 2, primary tumour sample 12 A was most dissimilar to the other samples, with a separate branch. In patient 3, the carcinomatosis sample 30 A was more similar to primary tumour sample 4B than to the other examined primary tumour regions. The same applies to patient 5, where the carcinomatosis sample 17 A clustered together with primary tumour sample 15 F. The coephenic coefficient for the cluster analysis for patient 3 was however only 0.32, indicating a weak clustering. Patient 4 had a number of carcinomatosis samples showing more similarity to each other than to the samples from the primary tumour and lymph node metastases. However, there was also a carcinomatosis sample (40 A) showing a higher similarity to a lymph node sample (62 A) than to samples from the primary tumour. The clustering analysis for patient 6 showed a similarity between carcinomatosis sample 23B and tumour sample 19 A and 20E, with primary tumour sample 15 J deviating from the others. In patient 7, carcinomatosis sample 29B, deposit sample 10B and 11 A, and liver metastasis samples 20 A and 21 A, showed the highest similarity to primary tumour samples 4 A and 5D. On the contrary, liver metastasis samples 22 A and 27 C, together with lymph node samples 9 C, 14 A and 38 A showed a higher similarity to primary tumour sample 4 K.

## Discussion

This study entails a thorough analysis of the genetic heterogeneity in colorectal peritoneal carcinomatosis, accompanied by comprehensive multi-region immuno-profiling to determine the expression of SATB2, a sensitive and highly specific marker for colorectal cancer, RBM3, a candidate prognostic and predictive biomarker, as well as a standard panel of mismatch repair proteins. It is, to the best of our knowledge, the hitherto most extensive study on this disease entity in terms of intra-patient sample size. Only patients with synchronous PC who had undergone CRS and HIPEC with curative intent, none of whom had received neoadjuvant treatment, were included. Thus, as all examined tumours were chemotherapy-naïve, the biomarker landscape had not been affected by treatment.

The distribution of the primary tumour location was even among the cases, with four having a tumour located in the proximal colon and three in the distal colon. Proximal location of the primary tumour is a known risk factor for PC^1^, hence, proximal tumours are more often seen in patients affected with PC than in patients with other types of disseminated CRC. Mucinous histology is also a known risk factor for PC^46^, and in this study, three out of the seven cases were classified as being mucinous or of signet-ring type. The PCI scores ranged between 4 and 24, and guidelines usually recommend CRS and HIPEC for patients with a PCI-score < 20. In our study, all patients with a PCI-score < 10 are still alive and disease-free, which supports previously published work showing a connection between PCI-score and survival^11,47^.

Immuno-profiling of individual TMAs, depicting the full extent of the disease, revealed some notable attributes, further supporting the notion that peritoneal carcinomatosis is an entity of its own. Of note, while tissue heterogeneity is frequently used as an argument against the use of TMAs, this argument is more valid in the high-throughput setting, where only a few tissue cores from multiple individual tumours are analyzed. The herein used approach, with each TMA representing multiple tissue cores from all available archival formalin-fixed paraffin-embedded blocks with primary tumours and metastases of sufficient size from individual patients, should provide a better overview of the degree of biomarker heterogeneity than single whole sections.

The SATB2 protein is expressed in a nearly tissue-specific fashion in the normal mucosa of the lower gastrointestinal tract^48^, and several studies have shown that loss of SATB2 is associated with more aggressive tumours and adverse clinical outcome^21,49^. In the present study, the expression of SATB2 was considerably lower than expected even in the metastatic setting. In a study by Mezheyeuski et al. only 6,8 % of the samples from 450 patients with mCRC lacked SATB2 expression^21^. SATB2 expression has been shown to be reduced in right-sided tumours^50^, but the anatomical location of the tumours cannot explain the striking sparsity of SATB2 expression in this study. Of note, the highest expression was seen in a right-sided tumour, and in particular within the metastatic burden. Therefore, these findings suggest that an overall low expression of SATB2 might indeed be a hallmark of mCRC with peritoneal carcinomatosis. This notion is also supported by the findings in the study by Mezheyeuski et al., who reported a lower expression of SATB2 in mCRC with peritoneal metastases compared to mCRC with other metastatic sites^21^. Moreover, even if SATB2 expression has been shown to be somewhat lower in mucinous adenocarcinoma (83%)^51^ and signet ring cell carcinoma (88%)^52^ compared to conventional adenocarcinoma, histology cannot explain the overall low SATB2 expression denoted in the present study.

Regarding RBM3 protein expression, previous studies have reported a largely consistent association between high expression and a favorable outcome, both in cohorts with mixed stages^53,54^, and in cohorts with mCRC only^18,55^. In the present study, the expression of RBM3 was all over low, which can be conceived as being in line with that expected in mCRC. RBM3 was also found to be heterogeneously expressed and often higher in lymph node or peritoneal metastases than in primary tumours, which is in line with our previous study on colorectal lung metastases^55^. However, and notably, the highest expression was observed in one of the cases with poor prognosis. While the small sample size does not allow for any firm conclusions to be drawn, this observation might also be indicative of a different biology in these tumours. Of note, contrasting associations of RBM3 expression with clinical outcome have been shown in particularly aggressive cancers such as pancreatic cancer^20^.

In the metastatic setting, only 5% of the CRC patients have tumours displaying dMMR^56^. In the present cohort, one case lacked expression of the MMR-proteins MLH1 and PSM2, and all samples from this case had a MSI score > 0.20, demonstrating an MSI phenotype. In this case, the primary tumour was located in the right colon, which is in line with previous studies showing dMMR to be more frequent in proximal colon cancer^57^. Furthermore, this case also displayed the highest TMB and the lowest concordance of mutations between the different samples. Willis et al. reported a correlation between MSI and discordance between tissue DNA and circulating tumour DNA-based mutation profiling^58^, suggesting MSI status to impel clonal evolution in mCRC. While the expression of MMR proteins MSH2 and MSH6 was homogenous across the samples from the case denoted as dMMR/MSI, expression of all MMR proteins was more heterogenous between the samples from the other patients, with some even being negative. Unfortunately, none of the tumour regions with negative MMR protein expression were of suitable quality for further analysis with TDS; hence no correlation between MMR protein expression and MSI-score could be made. The MSI-score for all other samples from the cases with proficient MMR were < 0.20, and thus denoted as MSS. Spatial heterogeneity of MMR-protein expression has previously been described in gastrointestinal cancer^59,60^, but the potential clinical implications have not been well studied. Despite the perfect concordance between MMR and MSI-score according to the currently established binary classification, this study highlights the importance of adequate sampling and extended analysis in future studies, addressing whether a more graded assessment of MMR protein expression may provide better predictive information, e.g., in the context of patient stratification for immunotherapy.

Our results, strictly based on analyses of chemotherapy-naïve tumour samples from patients with synchronous metastatic disease, also demonstrate an inter-patient heterogeneity regarding known driver mutations, which is in line with previously published data on CRC, with *TP53*, *APC,* and *KRAS* being the most frequently mutated genes^57,61^. A uniform distribution of mutations in *KRAS* and *BRAF* was further observed across samples from individual patients, which is also in line with previously published data^62^, and of clinical relevance, given their established role as predictive biomarkers for targeted therapies. Mutations in *TP53* were observed in three cases, two of which with concomitant mutations in *APC* and *KRAS*, indicating a tumour development in line with the tumourigenesis model described by Fearon et al.^63^. In two of the patients with sequencing variants in the *TP5*3 gene, a heterozygous copy number loss of the same gene was also observed in all or in the majority of the samples, indicating inactivation of both *TP53* alleles.

In one of the cases with a more aggressive disease course, a mutation in *PPP2R1A* was found in all samples. This mutation is rare in CRC, but is seen in around 40% of patients with endometrial cancer type II^64^. The *PPP2R1A* gene encodes for the phosphatase PP2A, which has shown to be a potential drug target^65^. The homogenous tissue distribution of the *PPP2R1A* mutation observed in this study further strengthens its suitability as a stable drug target. In another case, with a favourable outcome and where no universal, clinically relevant, mutations could be found, 10 of 14 samples had a *PIK3CA* mutation. The mutation was only seen in samples from the primary tumour and lymph node metastases. Some samples from the primary tumour and lymph node metastases were however also wild-type, as well as all PC samples. Literature on the potential spatial heterogeneity of *PIK3CA* mutations is sparse, but the issue merits further study, not least in light of ongoing trials on the efficacy of 5-ASA treatment in preventing relapse in *PIK3CA*-mutated CRC (ALASCCA study, NCT02647099).

Although the copy number analysis in the present study was primarily focused on specific gene targets, it highlights the presence of intra-patient as well as inter-patient copy number heterogeneity in CRC with peritoneal carcinomatosis. The importance of copy number heterogeneity, and its role in tumourigenesis, has been studied in other types of cancer and has been shown to impact patient survival^66–68^. Thus, the results from this study should encourage future in-depth studies with genome-wide mapping of the copy number landscape in colorectal peritoneal carcinomatosis, to further unravel the metastatic cascade.

The hierarchical clustering reveals some noteworthy differences between the patients. In patient 1, all lymph node samples were most closely linked to the same primary tumour, whereas in patient 2 and 4, multiple carcinomatosis samples showed higher similarity to the lymph nodes than to the samples from the primary tumour. The previously mentioned *peritoneal metastatic cascade* states that cells from the primary tumour detach and enter the peritoneal cavity, and that the attachment to the peritoneum can occur via lymphatic so called milky spots rich in vascular endothelial growth factor (VEGF)^26^. Of note, the performed clustering analysis does not provide information on the temporal aspect of the emergence of differences between two samples. It is however not unlikely that dissemination may occur from the primary tumour, via peritoneal carcinomatosis and then further on to lymph nodes or vice versa. However, we cannot rule out that a more extensive sampling from the primary tumour would provide a higher resolution of the pathways of dissemination. In patient 7, two main clusters were unveiled, both harbouring samples from liver metastases and the primary tumour. Of note, only one of the clusters comprised samples from peritoneal carcinomatosis and tumour deposits, whereas the other cluster included all lymph node samples. While it cannot be ruled out that some or all of the tumour deposits are in fact lymph node metastases overgrown by tumour, it can also be speculated that, in this case, the lymph nodes may be involved in the transmesothelial peritoneal dissemination process, instead of the translymphatic pathway earlier described, as proposed by Lemoine et al.^26^.

One patient, nr 7, also had distant metastases to the liver. Of the five examined liver metastasis samples, two and three, respectively, clustered together before clustering with samples of different origins. Zhang et al. investigated the dissemination route for liver metastases originating from CRC in three patients, and found that in one case, the liver metastases originated from the lymph node metastases rather than from the primary tumour^12^. But since the presence of distant metastases to other organs is, in general, a contraindication to undergo CRS and HIPEC procedure, it is difficult to perform larger studies on the intra-patient heterogeneity in this context.

In summary, comprehensive intra-patient molecular profiling of multiple samples from primary tumours, lymph node metastases, and peritoneal metastases indicates that mCRC with peritoneal metastases might, at least in some aspects, be a separate disease entity. This conclusion is drawn with the overall low expression of SATB2 and the clustering of PC to lymph node samples in mind. Larger studies, preferably carried out in the prospective setting, are needed to further explore clinically relevant biological traits and potential therapeutic targets.

## Supplementary information

Supplementary Figure 1

Supplementary table 1-3

## Data Availability

The datasets used and analyzed during the current study are available from the corresponding author on reasonable request.

## References

[1] Segelman J (2012). Incidence, prevalence and risk factors for peritoneal carcinomatosis from colorectal cancer. Br. J. Surg..

[2] Lemmens VE (2011). Predictors and survival of synchronous peritoneal carcinomatosis of colorectal origin: A population-based study. Int. J. Cancer.

[3] Jayne DG, Fook S, Loi C, Seow-Choen F (2002). Peritoneal carcinomatosis from colorectal cancer. Br. J. Surg..

[4] Sugarbaker PH (1988). Surgical management of peritoneal carcinosis: Diagnosis, prevention and treatment. Langenbecks Arch. Chir..

[5] Jafari MD (2014). Surgical outcomes of hyperthermic intraperitoneal chemotherapy: Analysis of the american college of surgeons national surgical quality improvement program. JAMA Surg..

[6] Foster JM (2019). Morbidity and mortality rates following cytoreductive surgery combined with hyperthermic intraperitoneal chemotherapy compared with other high-risk surgical oncology procedures. JAMA Netw. Open..

[7] Verwaal VJ, van Tinteren H, Ruth SV, Zoetmulder FA (2004). Toxicity of cytoreductive surgery and hyperthermic intra-peritoneal chemotherapy. J. Surg. Oncol..

[8] Cashin PH (2016). Cytoreductive surgery and intraperitoneal chemotherapy versus systemic chemotherapy for colorectal peritoneal metastases: A randomised trial. Eur. J. Cancer.

[9] Verwaal VJ (2003). Randomized trial of cytoreduction and hyperthermic intraperitoneal chemotherapy versus systemic chemotherapy and palliative surgery in patients with peritoneal carcinomatosis of colorectal cancer. J. Clin. Oncol.: Off J. Am. Soc. Clin. Oncol..

[10] Quenet F (2019). A UNICANCER phase III trial of Hyperthermic Intra-peritoneal Chemotherapy (HIPEC) for Colorectal Peritoneal Carcinomatosis. PRODIGE 7. Eur. J. Surg. Oncol..

[11] Elias D (2010). Peritoneal colorectal carcinomatosis treated with surgery and perioperative intraperitoneal chemotherapy: Retrospective analysis of 523 patients from a multicentric French study. J. Clin. Oncol..

[12] Zhang C (2020). Mapping the spreading routes of lymphatic metastases in human colorectal cancer. Nat. Commun..

[13] Kim R (2015). Co-evolution of somatic variation in primary and metastatic colorectal cancer may expand biopsy indications in the molecular era. PLoS One..

[14] Schweiger T (2018). Mutational profile of colorectal cancer lung metastases and paired primary tumors by targeted next generation sequencing: implications on clinical outcome after surgery. J. Thorac. Dis..

[15] Enblad M (2019). Gains of chromosome 1p and 15q are associated with poor survival after cytoreductive surgery and HIPEC for treating colorectal peritoneal metastases. Ann. Surg. Oncol..

[16] Van Cutsem E (2016). ESMO consensus guidelines for the management of patients with metastatic colorectal cancer. Ann. Oncol..

[17] André T (2020). Pembrolizumab in microsatellite-instability-high advanced colorectal cancer. N. Engl. J. Med..

[18] Siesing C (2017). High RBM3 expression is associated with an improved survival and oxaliplatin response in patients with metastatic colorectal cancer. PLoS One..

[19] Ehlen A (2010). Expression of the RNA-binding protein RBM3 is associated with a favourable prognosis and cisplatin sensitivity in epithelial ovarian cancer. J. Transl. Med..

[20] Karnevi E (2018). Translational study reveals a two-faced role of RBM3 in pancreatic cancer and suggests its potential value as a biomarker for improved patient stratification. Oncotarget.

[21] Mezheyeuski A (2020). Metastatic colorectal carcinomas with high SATB2 expression are associated with better prognosis and response to chemotherapy: A population-based Scandinavian study. Acta. Oncol..

[22] Welch DR (2016). Tumor heterogeneity–A ‘Contemporary Concept’ founded on historical insights and predictions. Cancer Res..

[23] Brannon AR (2014). Comparative sequencing analysis reveals high genomic concordance between matched primary and metastatic colorectal cancer lesions. Genome Biol..

[24] Dang HX (2020). The clonal evolution of metastatic colorectal cancer. Sci. Adv..

[25] Naxerova K (2017). Origins of lymphatic and distant metastases in human colorectal cancer. Sci. (N. Y, N. Y).

[26] Lemoine L, Sugarbaker P, Van der Speeten K (2016). Pathophysiology of colorectal peritoneal carcinomatosis: Role of the peritoneum. World J. Gastroenterol..

[27] Jayne, D. Molecular Biology of Peritoneal Carcinomatosis. In: Wim P. Ceelen (ed). *Peritoneal Carcinomatosis: A Multidisciplinary Approach*10.1007/978-0-387-48993-3_2 21–33 (Springer US: Boston, MA, 2007). 10.1007/978-0-387-48993-3_2 17633045

[28] Hau SO (2020). Chemotherapy, host response and molecular dynamics in periampullary cancer: The CHAMP study. BMC Cancer.

[29] Cancer, A. J. C. o. *AJCC CANCER STAGING MANUAL**Seventh Edition*. (Springer, 2010).

[30] Eurofins. https://eurofinsgenomics.eu/en/eurofins-genomics/product-faqs/next-generation-sequencing/questions-on-inview-oncopanel-all-in-one/ (2021).

[31] Li H, Durbin R (2009). Fast and accurate short read alignment with Burrows-Wheeler transform. Bioinformatics.

[32] Tarasov A, Vilella AJ, Cuppen E, Nijman IJ, Prins P (2015). Sambamba: fast processing of NGS alignment formats. Bioinformatics.

[33] DePristo MA (2011). A framework for variation discovery and genotyping using next-generation DNA sequencing data. Nat. Genet..

[34] McKenna A (2010). The Genome Analysis Toolkit: a MapReduce framework for analyzing next-generation DNA sequencing data. Genome Res..

[35] Wilm A (2012). LoFreq: a sequence-quality aware, ultra-sensitive variant caller for uncovering cell-population heterogeneity from high-throughput sequencing datasets. Nucleic Acids Res..

[36] Tate JG (2019). COSMIC: The catalogue of somatic mutations in cancer. Nucleic Acids Res..

[37] Talevich E, Shain AH, Botton T, Bastian BC (2016). CNVkit: Genome-wide copy number detection and visualization from targeted DNA sequencing. PLoS Comput. Biol..

[38] Allgäuer M (2018). Implementing tumor mutational burden (TMB) analysis in routine diagnostics-a primer for molecular pathologists and clinicians. Transl. Lung Cancer Res..

[39] Meléndez B (2018). Methods of measurement for tumor mutational burden in tumor tissue. Transl. Lung Cancer Res..

[40] Forbes SA (2015). COSMIC: Exploring the world’s knowledge of somatic mutations in human cancer. Nucleic Acids Res..

[41] Landrum MJ (2016). ClinVar: Public archive of interpretations of clinically relevant variants. Nucleic Acids Res..

[42] Sherry ST (2001). dbSNP: The NCBI database of genetic variation. Nucleic Acids Res..

[43] Lek M (2016). Analysis of protein-coding genetic variation in 60,706 humans. Nature.

[44] Sun JX (2018). A computational approach to distinguish somatic vs. germline origin of genomic alterations from deep sequencing of cancer specimens without a matched normal. PLoS Comput. Biol..

[45] Salipante SJ, Scroggins SM, Hampel HL, Turner EH, Pritchard CC (2014). Microsatellite instability detection by next generation sequencing. Clin. Chem..

[46] Hugen N, van de Velde CJH, de Wilt JHW, Nagtegaal ID (2014). Metastatic pattern in colorectal cancer is strongly influenced by histological subtype. Ann Oncol.

[47] Goéré D (2015). Extent of colorectal peritoneal carcinomatosis: attempt to define a threshold above which HIPEC does not offer survival benefit: a comparative study. Ann. Surg. Oncol..

[48] Magnusson K (2011). SATB2 in combination with cytokeratin 20 identifies over 95% of all colorectal carcinomas. Am. J. Surg. Pathol..

[49] Eberhard J (2012). A cohort study of the prognostic and treatment predictive value of SATB2 expression in colorectal cancer. Br. J. Cancer.

[50] Ma C (2019). SATB2 and CDX2 are prognostic biomarkers in DNA mismatch repair protein deficient colon cancer. Mod. Pathol..

[51] Ramos BD (2019). A comprehensive evaluation of special AT-rich Sequence-binding Protein 2 (SATB2) immunohistochemical staining in mucinous tumors from gastrointestinal and nongastrointestinal sites. Appl. Immunohistochem. Mol. Morphol..

[52] Ma C, Lowenthal BM, Pai RK (2018). SATB2 Is superior to CDX2 in distinguishing signet ring cell carcinoma of the upper gastrointestinal tract and lower gastrointestinal tract. Am. J. Surg. Pathol..

[53] Hjelm B (2011). High nuclear RBM3 expression is associated with an improved prognosis in colorectal cancer. Proteom. Clin. Appl..

[54] Melling N (2015). RBM3 expression loss is associated with right-sided localization and poor prognosis in colorectal cancer. Histopathology.

[55] Vidarsdottir H (2021). Clinical significance of RBM3 expression in surgically treated colorectal lung metastases and paired primary tumors. J. Surg. Oncol..

[56] Venderbosch S (2014). Mismatch repair status and BRAF mutation status in metastatic colorectal cancer patients: A pooled analysis of the CAIRO, CAIRO2, COIN, and FOCUS studies. Clin. Cancer Res..

[57] The Cancer Genome Atlas Network (2012). Comprehensive molecular characterization of human colon and rectal cancer. Nature..

[58] Willis J (2018). Impact of microsatellite instability (MSI) on tumor clonal evolution in metastatic colorectal cancer (mCRC). J. Clin. Oncol..

[59] Joost P (2014). Heterogenous mismatch-repair status in colorectal cancer. Diagn. Pathol..

[60] McCarthy AJ (2019). Heterogenous loss of mismatch repair (MMR) protein expression: A challenge for immunohistochemical interpretation and microsatellite instability (MSI) evaluation. J. Pathol. Clin. Res..

[61] Mendelaar PAJ (2021). Whole genome sequencing of metastatic colorectal cancer reveals prior treatment effects and specific metastasis features. Nat. Commun..

[62] Kim TM (2015). Subclonal genomic architectures of primary and metastatic colorectal cancer based on intratumoral genetic heterogeneity. Clin. Cancer Res..

[63] Fearon ER, Vogelstein B (1990). A genetic model for colorectal tumorigenesis. Cell..

[64] Remmerie M, Janssens V (2019). PP2A: A promising biomarker and therapeutic target in endometrial cancer. Front. Oncol..

[65] Vainonen JP, Momeny M, Westermarck J (2021). Druggable cancer phosphatases. Sci. Transl. Med..

[66] Andersson N (2020). Extensive clonal branching shapes the evolutionary history of high-risk pediatric cancers. Cancer Res..

[67] Turajlic S (2018). Deterministic evolutionary trajectories influence primary tumor growth: TRACERx Renal. Cell..

[68] Jamal-Hanjani M (2017). Tracking the evolution of non-small-cell lung cancer. N. Engl. J. Med..

