# Supplementary figures and images for: Delineating the intra-patient heterogeneity of molecular alterations in treatment-naïve colorectal cancer with peritoneal carcinomatosis
Source: Mod Pathol. 2022 Feb 15;35(7):979–88. doi: 10.1038/s41379-022-01012-y (PMC9249627; doi:10.1038/s41379-022-01012-y)

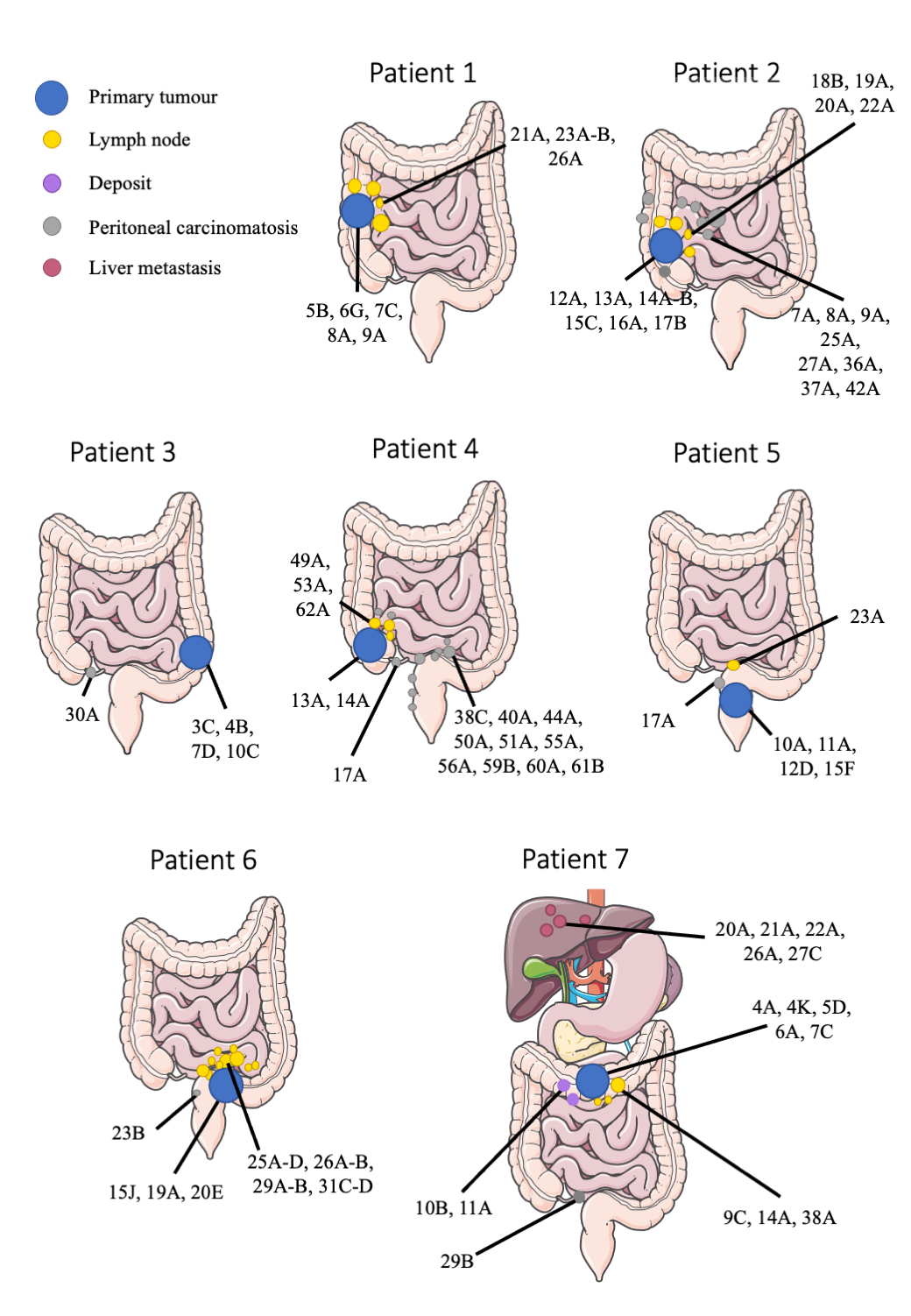

Supplement: Supplementary file 1 — Supplementary Figure 1 [file 41379_2022_1012_MOESM1_ESM.tif]
